# Antimicrobial and Anti-Virulence Activity of Capsaicin Against Erythromycin-Resistant, Cell-Invasive Group A Streptococci

**DOI:** 10.3389/fmicb.2015.01281

**Published:** 2015-11-13

**Authors:** Emanuela Marini, Gloria Magi, Marina Mingoia, Armanda Pugnaloni, Bruna Facinelli

**Affiliations:** ^1^Unit of Microbiology, Department of Biomedical Sciences and Public Health, Polytechnic University of Marche, Ancona, Italy; ^2^Department of Clinical and Molecular Sciences, Polytechnic University of Marche, Ancona, Italy

**Keywords:** capsaicin, Group A streptococci, virulence, biofilm, haemolytic activity, cell invasion, hormesis

## Abstract

Capsaicin (8-methyl-N-vanillyl-6-nonenamide) is the active component of *Capsicum* plants (chili peppers), which are grown as food and for medicinal purposes since ancient times, and is responsible for the pungency of their fruit. Besides its multiple pharmacological and physiological properties (pain relief, cancer prevention, and beneficial cardiovascular, and gastrointestinal effects) capsaicin has recently attracted considerable attention because of its antimicrobial and anti-virulence activity. This is the first study of its *in vitro* antibacterial and anti-virulence activity against *Streptococcus pyogenes* (Group A streptococci, GAS), a major human pathogen. The test strains were previously characterized, erythromycin-susceptible (*n* = 5) and erythromycin-resistant (*n* = 27), cell-invasive pharyngeal isolates. The MICs of capsaicin were 64–128 μg/mL (the most common MIC was 128 μg/mL). The action of capsaicin was bactericidal, as suggested by MBC values that were equal or close to the MICs, and by early detection of dead cells in the live/dead assay. No capsaicin-resistant mutants were obtained in single-step resistance selection studies. Interestingly, growth in presence of sublethal capsaicin concentrations induced an increase in biofilm production (*p* ≤ 0.05) and in the number of bacteria adhering to A549 monolayers, and a reduction in cell-invasiveness and haemolytic activity (both *p* ≤ 0.05). Cell invasiveness fell so dramatically that a highly invasive strain became non-invasive. The dose-response relationship, characterized by opposite effects of low and high capsaicin doses, suggests a hormetic response. The present study documents that capsaicin has promising bactericidal activity against erythromycin-resistant, cell-invasive pharyngeal GAS isolates. The fact that sublethal concentrations inhibited cell invasion and reduced haemolytic activity, two important virulence traits of GAS, is also interesting, considering that cell-invasive, erythromycinresistant strains can evade β-lactams by virtue of intracellular location and macrolides by virtue of resistance, thus escaping antibiotic treatment. By inhibiting intracellular invasion and haemolytic activity, capsaicin could thus prevent both formation of a difficult to eradicate intracellular reservoir, and infection spread to deep tissues.

## Introduction

The global burden of antibiotic resistance has revived the interest in the antimicrobial properties of plants (Cowan, 1999; Hemaiswarya et al., 2008; Hyldgaard et al., 2012). Capsaicin (8-methyl-N-vanillyl-6-nonenamide) is the active component of *Capsicum* plants (chili peppers), which are grown as food and for medicinal purposes since ancient times, and is responsible for the pungency of their fruit (Cichewicz and Thorpe, 1996). Capsaicin and related compounds (called capsaicinoids) are secondary metabolites of chili peppers that play an important role in plant defense, probably as repellents against animals (Jensen et al., 2003). Besides its multiple pharmacological and physiological properties (pain relief, cancer prevention, beneficial cardiovascular, and gastrointestinal effects; Luo et al., 2011), capsaicin has recently attracted considerable attention because of its antimicrobial and anti-virulence activity. A bactericidal effect has been described against food-borne pathogens, *Helicobacter pylori*, and *Pseudomonas aeruginosa* (Cowan, 1999; Omolo et al., 2014), whereas an anti-virulence activity has been demonstrated against *Vibrio cholerae*, *Staphylococcus aureus*, and *Porphyromonas gingivalis* (Chatterjee et al., 2010; Kalia et al., 2012; Qiu et al., 2012; Zhou et al., 2014).

*Streptococcus pyogenes* (Group A streptococci, GAS) is a major human pathogen with a high prevalence worldwide (Bisno et al., 2003; Cunningham, 2008). Clinical manifestations range from non-invasive, self-limiting purulent infections of the pharynx and skin to severe, invasive infections such as necrotizing fasciitis, sepsis, toxic shock-like syndrome; sequelae include acute rheumatic fever, rheumatic heart disease, and glomerulonephritis (Cunningham, 2008). GAS are the most common cause of acute bacterial pharyngotonsillitis in children (Logan et al., 2012). Its multiple virulence factors enable it to attach to host tissues, evade the host immune response, invade cells, and spread by penetrating tissue layers (Bisno et al., 2003; Cunningham, 2008). Virulence factors include streptolysin S, a potent cytolytic toxin that contributes to deep tissue invasion and is responsible for the haemolytic zone around colonies grown on blood agar plates (Sumitomo et al., 2011); the fibronectin binding protein F1 (encoded by gene *prtF1*), an adhesin required for efficient entry into epithelial cells (Cunningham, 2008); and the ability to form biofilm (Fiedler et al., 2015). Biofilms are surfaceassociated bacterial communities embedded in a self-produced polysaccharide matrix that allow bacteria to persist *in vivo* by resisting both host immune defenses and antibiotics (Fiedler et al., 2015).

Although GAS are uniformly susceptible to β-lactams, a general increase in resistance to macrolides—due to the presence of macrolide efflux (*mef*) and erythromycin methylase (*erm*) genes—has been reported over the past few decades in several areas of the world (Giovanetti et al., 2002; Kaplan and Cornaglia, 2005; Gracia et al., 2009; Liang et al., 2012). β-lactams (particularly penicillin) are the drugs of choice to treat GAS pharyngotonsillitis, whereas macrolides are used in individuals with penicillin allergy. However, unlike macrolides, β-lactams have little effect on intracellular bacteria, which may be a reason for the failure of penicillin (Gillespie, 1998; Cunningham, 2008). Therefore, strains combining macrolide resistance and an ability to enter into human respiratory cells may escape β-lactams by virtue of their intracellular location and macrolides by virtue of resistance (Facinelli et al., 2001). Biofilm formation further hampers antibiotic treatment and eradication (Baldassarri et al., 2006).

In this study, we first evaluated the antibacterial and anti-virulence properties of capsaicin against previously characterized, cell-invasive (*prtF1*-positive), clinical GAS strains, all isolated in Italy from children with pharyngotonsillitis.

## Materials and Methods

### GAS Strains and Growth Media

A total of 32 GAS isolates, including 27 erythromycin-resistant [minimum inhibitory concentration (MIC) ≥ 1 μg/mL] and 5 erythromycin-susceptible strains isolated throughout Italy from children with pharyngitis (Varaldo et al., 1999), were examined. All strains had previously been characterized (Facinelli et al., 2001; Spinaci et al., 2004, 2006) in terms of erythromycin resistance phenotype/genotype [*erm*(B)/cMLS (*n* = 6); *erm*(B)/iMLS (*n* = 5); *erm*(TR)/iMLS (*n* = 6); *mef*(A)/M (*n* = 10)]; *emm* type (12 different *emm* types); the presence of the *prtF1* gene, and cell invasiveness. Each of the 32 strains is a clone identified among Italian GAS isolates.

Blood agar base (BAB) supplemented with 5% sheep blood, Müller-Hinton agar (MHA) supplemented with 5% sheep blood, Müller-Hinton cation-adjusted broth (CAMHB) supplemented with 3% laked sheep blood, brain heart infusion (BHI) agar and broth, Todd-Hewitt broth (THB) and Tryptone Soya Broth (TSB), all from Oxoid (Basingstoke, UK) were used throughout the study. Isolates were maintained in glycerol at –70°C and subcultured twice on BAB before testing.

### Susceptibility Tests

Capsaicin (M2028, ≥ 95.0% purity) was purchased from Sigma–Aldrich (St. Louis, MO, USA) and stored (10 mg/mL stock solution) in absolute ethanol at –20°C. The MIC, i.e., the lowest concentration of capsaicin that inhibited the visible growth of streptococci after overnight incubation, was determined in blood-supplemented CAMHB by the microdilution method, as recommended by Clinical and Laboratory Standards Institute (CLSI) guidelines (Clinical and Laboratory Standards Institute, 2015). The minimum bactericidal concentration (MBC) was determined by plating 10 μL of each microdilution on blood-supplemented MHA followed by overnight incubation at 37°C in 5% CO_2_. The MBC was defined as the lowest concentration of capsaicin that killed 99.9% of the inoculum. All experiments were performed in triplicate.

### Live/dead Assay

Survival in presence of capsaicin was tested by the live/dead assay using SYBR Green I (Invitrogen, Eugene, OR, USA) and propidium iodide (Sigma–Aldrich), two nucleic acid dyes differing in their ability to penetrate bacterial cells (Magi et al., 2015). Briefly, overnight-grown streptococci were suspended in 1 mL capsaicin-supplemented BHI broth [∼1 × 10^8^ colony forming units (CFU)/mL] and incubated for 15, 30, or 60 min at 37°C in 5% CO_2_. After staining with 1 × SYBR Green I and 40 μg/mL propidium iodide, cells were incubated at room temperature for 25 min in the dark, harvested on GTBP filters (Ø = 0.2 μm, Millipore, Billerica, MA, USA), and examined under an epifluorescence microscope (Axioskop2, Zeiss, Milano, Italy).

### Search for Capsaicin-resistant Mutants

Experiments were performed as reported previously (Magi et al., 2015). Briefly, bacterial cells were grown overnight in BHI agar plates, scraped off, washed once with BHI broth, and resuspended to a final concentration of 1 × 10^10^ to 1 × 10^11^ CFU/mL. Then, 100 μL of the bacterial suspension was spread on capsaicin-containing BAB plates at 1, 2, and 4 times the MIC. The plates were incubated at 37°C in CO_2_ for 72 h. Experiments were repeated twice.

### Biofilm Formation Assay

Biofilm formation was tested as recently described by Vignaroli et al. (2013). Briefly, bacteria were grown overnight in 2 mL TSB containing 1% glucose at 37°C in 5% CO_2_. Overnight bacterial suspensions were prepared to yield final inocula of ∼1 × 10^8^ CFU/mL; then 200 μL aliquots of the bacterial suspension was inoculated into 96-well microtiter plates at least in triplicate. After overnight incubation at 37°C in 5% CO_2_, wells were washed three times in phosphate-buffered saline (PBS), dried for 1 h at 60°C, and stained with Hucker’s crystal violet. After three washes in sterile water, wells were inoculated with 100 μL of 95% ethanol and shaken for 10 min. Biofilm formation was then quantified by measuring absorbance at 690 nm with a Multiscan Ascent apparatus (Thermo Scientific, Waltham, MA, USA). The optical density (OD) cut-off (ODc) was defined as three standard deviations above the mean OD of the negative control, represented by non-inoculated wells containing TSB (Stepanovic et al., 2000). Strains were classified as non-producer (OD ≤ ODc), weak producer (ODc < OD ≤ 2 × ODc), or strong producer (OD > 4 × ODc). The biofilm-forming strain *S. epidermidis* ATCC 35984 was used as a positive control (Christensen et al., 1985).

In some experiments, biofilm formation was evaluated in presence of sublethal capsaicin concentrations (1/4, 1/8, and 1/16 × MIC). Briefly, overnight bacterial suspensions were prepared to yield final inocula of ∼2 × 10^8^ CFU/mL. Then, 100 μL of bacterial culture and 100 μL of different sub-MIC capsaicin concentrations were added to each well of a 96-well microplate. Wells containing 100 μL of the bacterial suspension and 100 μL of TSB without capsaicin were used as positive controls. After incubation, biofilm formation was evaluated as described above.

All experiments were performed in triplicate.

### Adhesion and Invasion Assays

The human alveolar carcinoma cell line A549 (ATCC CCL 185) was used in all experiments. Cells were routinely cultured in RPMI 1640 supplemented with 1% (v/v) L-glutamine and 10% (v/v) fetal calf serum (all from Gibco, Grand Island, NY, USA) in 50 mL (25 cm^2^) plastic tissue culture flasks (Corning Costar, Milano, Italy) at 37°C in an atmosphere containing 5% CO_2_. Cell monolayers were trypsinized and adjusted to a concentration of 2.5 × 10^5^ cells/mL in culture medium; 1 mL cell suspension was dispensed into each 22-mm well of a 12-well tissue culture plate and incubated to obtain confluent monolayers (Facinelli et al., 2001).

Adhesion and invasion experiments were performed using GAS strains grown in presence of sublethal capsaicin concentrations (1/4, 1/8, and 1/16 × MIC). Briefly, after overnight growth in blood agar plates, streptococci were grown in THB for 6–8 h at 37°C, diluted 1:100 in fresh broth containing capsaicin sub-MICs (1/4, 1/8, and 1/16 × MIC), and incubated for 16 h at 37°C. Bacterial cells were harvested by centrifugation; the pellets were resuspended in PBS and re-centrifuged. The final pellets were resuspended in RPMI 1640 (with 10% fetal calf serum and 1% L-glutamine) and capsaicin sub-MIC, and incubated for 1 h at 37°C. After 3 washes with PBS, monolayers were infected with 1–3 × 10^5^ CFU/mL, suspended in RPMI 1640 without serum, and incubated for 2 h at 37°C in 5% CO_2_.

To evaluate adherent bacteria, infected monolayers were washed 3 times with PBS and lysed with cold distilled water. Aliquots of the appropriate dilutions of the lysate were plated in BHI agar and incubated overnight at 37°C, to count total adherent bacteria per mL (CFU/mL). To determine viable intracellular bacteria, infected monolayers were washed 3 times with PBS and covered with 2 mL RPMI 1640 containing bactericidal concentrations of penicillin (5 μg/mL) and gentamicin (100 μg/mL). After 2 h at 37°C in 5% CO_2_, monolayers were washed and lysed as described above, then viable internalized bacteria (CFU/mL) were counted by plating the lysates on BHI agar. Each assay represented the average of triplicate wells. All experiments were performed twice.

For microscopic observation, monolayers were infected as described above. After washes with PBS, cells were fixed with methanol for 10 min, stained with 20% Giemsa for 20 min (Darfeuille-Michaud et al., 1998), and finally examined with a Leica DMRB microscope (Leica Microsystems, Wetzlar, Germany) using a 100 × oil-immersion objective.

### Determination of Cell Viability

The effect of capsaicin on A549 cell monolayers was evaluated by the trypan blue exclusion method (Facinelli et al., 1998). Briefly, monolayers were grown on SlideFlask and then incubated with capsaicin sub-MICs (1/4, 1/8, and 1/16 × MIC) for 15, 30, and 60 min. Control cultures were incubated without capsaicin. At the end of the incubation period, monolayers were washed, stained with 0.4% Trypan Blue solution (Gibco) at room temperature for 30 min, and examined under a light microscope at 20x magnification. The viable cell ratio (%) was calculated as the number of unstained cells/total cell number × 100.

### Haemolysis Assay

Haemolytic activity was measured as described previously (Smith-Palmer et al., 2004). Briefly, 1 mL of culture supernatant of test strains grown overnight in presence of capsaicin sub-MICs (1/4, 1/8, and 1/16 × MIC) was incubated with 485 μL PBS, to which sheep erythrocytes were added to achieve a final concentration of 1%; 100% haemolysis was obtained with 1% erythrocytes in water. Samples were incubated at 37°C for 30 min, centrifuged (1000 × *g*) for 5 min, and read by a spectrophotometer at a wavelength of 690 nm. Results were expressed as percentage of haemolysis with respect to the control strain, grown without capsaicin. All experiments were performed in triplicate.

### Time-kill Curves

Time-kill experiments were performed in microtiter plates containing different capsaicin sub-MICs (1/4, 1/8, and 1/16 × MIC) in BHI broth, as described previously (Magi et al., 2015). Briefly, streptococci (∼5 × 10^5^ CFU/mL) were placed on microtiter plates, incubated for 24 h at 37°C and read at OD_690_ at 1-h intervals using a Multiscan Ascent apparatus. Controls were grown in absence of capsaicin. All experiments were performed in triplicate.

### Statistical Analysis

Differences between groups were assessed with paired Student’s *t*-test using GraphPad software. Values are reported as mean ± standard deviation (SD). *P*-values ≤ 0.05 were considered statistically significant.

## Results

The susceptibility studies involved all 32 strains. The capsaicin MICs of erythromycin-resistant and erythromycin-susceptible strains were prevalently included in a narrow range (64–128 μg/mL), where the most common MIC was 128 μg/mL (23/32 strains); one strain exhibited a MIC of 512 μg/mL. MBCs were equal or close (one dilution) to the MICs.

**FIGURE 1:**
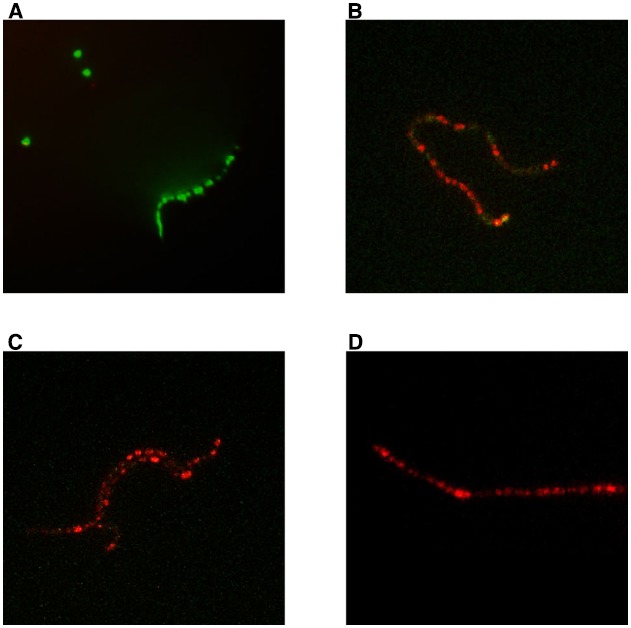
**Bactericidal action of capsaicin against strain SP1070.** Live/dead assay: **(A)** control, no capsaicin; **(B)** 15 min incubation, **(C)** 30 min incubation, and **(D)** 60 min incubation in presence of capsaicin MIC (128 μg/mL). Bacteria with intact cell membranes stain fluorescent green, those with damaged membranes stain fluorescent red.

In the live/dead assay, using strain SP1070 and capsaicin at the MIC (128 μg/mL), several red cells were detected as early as 15 min and all cells were red after 60 min (Figure 1).

No capsaicin-resistant mutants were found in single-step resistance selection experiments using strains SP1070, 9713, and SP114 (capsaicin MIC = 128 μg/mL).

Biofilm production in presence of sublethal capsaicin concentrations was evaluated using strong (SP55, 9713, 1814, SP1070, SP114, and 152–006) and weak (9408 and 68–006) biofilm producers. Biofilm production increased in all strains from 12 to 255%, maximum biofilm production occurring at 8 and 16 μg/mL (strong producers), and at 16 and 32 μg/mL (weak producers; Table 1). At a concentration of 64 μg/mL (1/2 × MIC), biofilm production fell from 4 to 97% in all strains.

**TABLE 1:** **Biofilm production by test strains in presence of capsaicin sub-MICs**.

	**OD_690_ at capsaicin sub-MICs (µg/mL)**			
**GAS strains**	**Control**	**1/16 (8)**	**1/8 (16)**	**1/4 (32)**
9408	0.25 ± 0.06	0.41 ± 0.06* (+64%)	0.44 ± 0.13* (+76%)	0.40 ± 0.05* (+60%)
68–006	0.26 ± 0.04	0.32 ± 0.03* (+23%)	0.33 ± 0.04* (+27%)	0.36 ± 0.040* (+39%)
SP1070	0.34 ± 0.06	0.45 ± 0.04*(+32%)	0.52 ± 0.05* (+53%)	0.42 ± 0.03* (+24%)
SP114	0.48 ± 0.16	0.80 ± 0.25 (+67%)	0.96 ± 0.09* (+100%)	0.73 ± 0.14* (+52%)
SP55	0.72 ± 0.23	0.92 ± 0.19* (+28%)	0.78 ± 0.18 (+8%)	0.81 ± 0.19 (+13%)
9713	0.97 ± 0.18	3.44 ± 0.14* (+255%)	2.92 ± 0.55* (+201%)	2.08 ± 0.20* (+114%)
152–006	1.00 ± 0.17	1.34 ± 0.34 (+34%)	2.14 ± 0.44* (+114%)	1.12 ± 0.16 (+12%)
1814	1.43 ± 0.31	3.85 ± 1.11* (+170%)	3.28 ± 1.11* (+129%)	1.74 ± 0.14* (+22%)

Each value represents mean OD_690_ ± SD of three experiments. The percent increases of biofilm formation also reported. Asterisks denote significant values with respect to the control (p ≤ 0.05). *p ≤ 0.05 with respect to the control.

**FIGURE 2:**
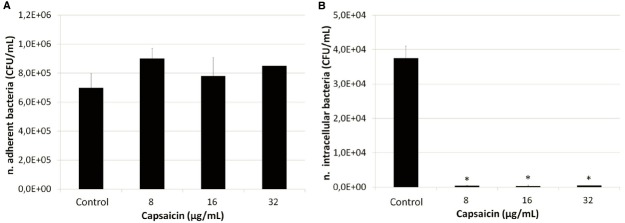
**Adhesion/invasion of A549 cells by strain SP1070 grown in presence of capsaicin sub-MICs. (A)** adhesion; **(B)** invasion. Results are the mean of adherent or intracellular streptococci (CFU/mL ± SD) of two experiments. Asterisks denote significant values with respect to the control (*p* ≤ 0.05).

**FIGURE 3:**
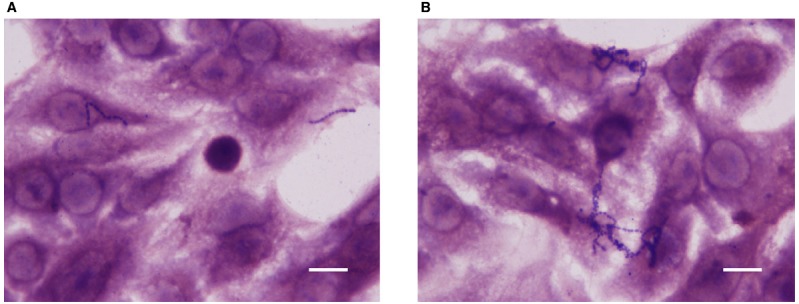
**Giemsa-stained A549 monolayers infected with strain SP1070 grown in presence of capsaicin sub-MICs.** Streptococci adherent to A549 monolayers in the absence **(A)** and presence of 8 μg/mL of capsaicin (1/16 × MIC) **(B)**. (Magnification: × 1000; scale bar: 10 μm).

Cell adhesion and invasion experiments were performed using the highly invasive strain SP1070. Remarkably, an increased number of bacteria adherent to A549 monolayers and a strong reduction in the number of intracellular bacteria were observed in presence of sublethal capsaicin concentrations (Figure 2). The increase in cell adhesion was also documented by Giemsa staining (Figure 3). The trypan blue exclusion test demonstrated 100% cell survival in both capsaicin-treated and control monolayers (data not shown).

**FIGURE 4:**
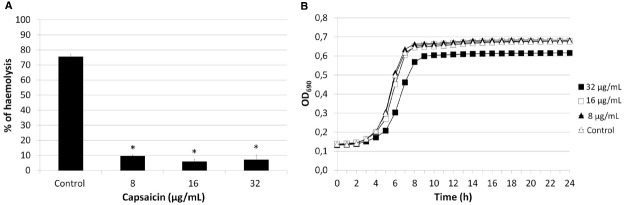
**Haemolytic activity and growth of strain SP1070 in presence of capsaicin sub-MICs. (A)** Percent haemolysis of SP1070 supernatants. Results are the mean ± SD of three experiments. Asterisks denote significant values with respect to the control (*p* ≤ 0.05). **(B)** Time-kill curve of SP1070 in presence of capsaicin sub-MICs.

Assessment of the haemolytic activity of strains SP1070, 9713, and SP114, grown in presence of sublethal capsaicin concentrations demonstrated a significant (*p* ≤ 0.05), dose-dependent decrease (29.28 to 92.03%) of haemolytic activity in all strains that was more evident in strain SP1070 (Figure 4A). In time–kill kinetic studies, the growth curve of strain SP1070 was not affected by sublethal capsaicin concentrations (Figure 4B).

## Discussion

In this study, we evaluated the antibacterial and anti-virulence properties of capsaicin against previously characterized cell-invasive pharyngeal GAS isolates.

Capsaicin was active against all isolates, both erythromycin-resistant and erythromycin-susceptible, even though the MICs were higher than those reported in recent studies using DMSO as the solvent (Nascimento et al., 2014). Our MICs might also have been lower using DMSO, but its intrinsic bactericidal activity ruled out its adoption (data not shown).

Capsaicin exerted a bactericidal action against streptococci, as suggested by the fact that the MBC values were equal to or one or two dilutions greater than the MICs. The bactericidal action of capsaicin was also assessed by the live/dead assay, where bacteria with intact cell membranes stain fluorescent green, and those with damaged membranes stain fluorescent red. The assay confirmed that capsaicin exerted a bactericidal action, probably through membrane damage, as suggested by the early detection of several red cells. A similar effect has recently been demonstrated by our group for carvacrol, a component of the essential oils of thyme and oregano (Magi et al., 2015). The present findings agree with previous studies documenting a bactericidal *in vitro* action of capsaicin on Gram-positive and Gram-negative pathogens (Omolo et al., 2014). Attempts to select for capsaicin-resistant mutants were unsuccessful, suggesting that capsaicin is not prone to develop resistance in GAS. Low-dose capsaicin exposure did not affect cell viability, demonstrating the lack of a cytotoxic effect.

The ability of low capsaicin concentrations to reduce bacterial virulence has recently been demonstrated *in vitro* in both Gram-positives and Gram-negatives (Xu et al., 2005; Chatterjee et al., 2010; Kalia et al., 2012; Qiu et al., 2012; Zhou et al., 2014). In this study, we evaluated the effects of exposure to capsaicin sub-MICs on some virulence traits of GAS. After strain growth in presence of sublethal concentrations, biofilm formation and adhesion to A549 epithelial cells were significantly enhanced. Interestingly, the decrease in cell invasiveness was so dramatic that a highly invasive strain became non-invasive. These data suggest that streptococci trapped in the biofilm matrix are no longer capable to invade cells, as hypothesized by Baldassarri et al. (2006). The effects induced by capsaicin on biofilm formation and adhesiveness are similar to those of chemically different antibiotics such as aminoglycosides, which at sub-MICs are able to induce biofilm formation and increase bacterial adherence to eukaryotic cells (Lorian, 1975; Kaplan, 2011; Andersson and Hughes, 2014). Moreover, the statistically significant reduction in haemolytic activity was not related to bacterial viability, as demonstrated by time–kill curves.

Successful establishment of GAS infection requires adhesion to host cells, colonization and, in certain cases, cell invasion followed by intracellular multiplication, dissemination, and/or persistence (Pizarro-Cerdá and Cossart, 2006). The present study documents that capsaicin has a promising bactericidal activity against erythromycin-resistant, cell-invasive pharyngeal GAS isolates. Moreover, sublethal concentrations inhibited cell invasion and reduced haemolytic activity, two important virulence traits of GAS. This is an interesting property, considering that cell-invasive, erythromycin-resistant strains are able to evade β-lactams by virtue of intracellular location and macrolides by virtue of resistance, thus escaping antibiotic treatment. By inhibiting intracellular invasion and haemolytic activity, capsaicin could thus prevent both formation of an intracellular reservoir that is difficult to eradicate, and infection spread to deep tissues. The anti-virulence properties of capsaicin have recently been reported. In particular, capsaicin at sublethal concentrations inhibits production of cholera toxin in *V. cholerae* and of alpha-toxin in *S. aureus* (Chatterjee et al., 2010; Qiu et al., 2012); it reduces *S. aureus* intracellular invasion (Kalia et al., 2012); and inhibits biofilm formation by *P. gingivalis* (Xu et al., 2005; Zhou et al., 2014). Our findings document a dose-response relationship characterized by contrasting effects of low and high capsaicin doses, suggesting a hormetic response (Kendig et al., 2010). Hormesis indicates biological responses—to environmental signals or stress stimuli—that are characterized by biphasic dose-response relationships, i.e., low-dose stimulation and high-dose inhibition (Kendig et al., 2010). Some antibiotics display dose-response relationships consistent with hormesis (Davies et al., 2006; Kendig et al., 2010); low-dose effects are well documented for penicillin, which at subinhibitory concentrations induces multiple non-lethal effects (e.g., morphological changes at the cell surface and induction of transcriptional and translational activities) that differ from the inhibitory effects observed at higher concentrations (Kendig et al., 2010).

Further evaluation in *in vivo* systems is required to determine whether the present findings can be exploited in treating GAS infections. Indeed, by inhibiting intracellular invasion and haemolytic activity, capsaicin could serve as a novel therapeutic tool against GAS infections, also preventing formation of an intracellular reservoir. The relationship between biofilm formation and reduced invasiveness and the molecular basis of the reduction of haemolytic activity are areas for future research.

### Conflict of Interest Statement

The authors declare that the research was conducted in the absence of any commercial or financial relationships that could be construed as a potential conflict of interest.
